# Three-dimensional Sponges with Super Mechanical Stability: Harnessing True Elasticity of Individual Carbon Nanotubes in Macroscopic Architectures

**DOI:** 10.1038/srep18930

**Published:** 2016-01-06

**Authors:** Zhaohe Dai, Luqi Liu, Xiaoying Qi, Jun Kuang, Yueguang Wei, Hongwei Zhu, Zhong Zhang

**Affiliations:** 1 CAS Key Laboratory of Nanosystem and Hierachical Fabrication, National Center for Nanoscience and Technology, Beijing 100190, China; 2 Center for Nano and Micro Mechanics (CNMM), Tsinghua University, Beijing 100084, China; 3 State Key Laboratory of Nonlinear Mechanics, Institute of Mechanics, Chinese Academy of Sciences, Beijing 100190, China; 4 CAS Key Laboratory of Mechanical Behavior and Design of Materials, Department of Modern Mechanics, University of Science and Technology of China, Hefei, Anhui 230027, China; 5 University of Chinese Academy of Science, Beijing 100049, China

## Abstract

Efficient assembly of carbon nanotube (CNT) based cellular solids with appropriate structure is the key to fully realize the potential of individual nanotubes in macroscopic architecture. In this work, the macroscopic CNT sponge consisting of randomly interconnected individual carbon nanotubes was grown by CVD, exhibiting a combination of super-elasticity, high strength to weight ratio, fatigue resistance, thermo-mechanical stability and electro-mechanical stability. To deeply understand such extraordinary mechanical performance compared to that of conventional cellular materials and other nanostructured cellular architectures, a thorough study on the response of this CNT-based spongy structure to compression is conducted based on classic elastic theory. The strong inter-tube bonding between neighboring nanotubes is examined, believed to play a critical role in the reversible deformation such as bending and buckling without structural collapse under compression. Based on *in-situ* scanning electron microscopy observation and nanotube deformation analysis, structural evolution (completely elastic bending-buckling transition) of the carbon nanotubes sponges to deformation is proposed to clarify their mechanical properties and nonlinear electromechanical coupling behavior.

Man-made cellular materials with porous structure, low density, large specific area and high damping capacity, have been increasingly developed for insulation, cushioning, buoyancy, filtering, catalyst support, sound absorbing and tissue scaffold applications^1^,^2^,^3^,^4^. Most familiar are the polymeric foams used in everything from ear plugs to crash pads of aircraft cockpit. Many of applications require that the materials have mechanical stability including resilience, load-bearing capacity, fatigue resistance and thermo-mechanical stability, while the stability performance of polymeric foams is limited by their temperature and time-dependent viscoelastic behavior such as creep and stress relaxation^5^,^6^. Even though a broad range of materials has been developed to meet various demands over the past decades, it remains a great challenge to design and fabricate the cellular solids with super mechanical stability. Recent works have highlighted the potential in development of macroscopic three-dimensional (3D) architectures from nanoscale building blocks for energy absorption, cushioning and flexible electronic devices^7^,^8^,^9^,^10^,^11^,^12^,^13^. Besides that, the multi-functionalities of nanofiller constituents would also widen the range of the man-made cellular solids and their diversity of applications^14^,^15^,^16^,^17^.

Among a wide range of nano-sized building blocks in different dimensionalities available, carbon nanotubes (CNTs) are extremely attractive due to their fascinating properties such as specific fibrous structure, marvelous tensile strength, excellent thermal stability, low density, electrical conductivity and particularly super-elasticity^18^,^19^,^20^,^21^. In fact, CNT-based sponge-like solids have demonstrated multi-functionality, good compressibility and ultra-light weight indeed, whereas the super mechanical stability is far from theoretical expectation. Aligned CNT arrays have shown remarkable mechanical resilience by utilizing the elasticity of individual CNTs under compression, whereas the entangled adjacent nanotubes within the aligned forest would cause the apparent decrease in stress during compressive cycles^7^,^8^,^22^. Recently, CNT-based cellular solids such as aerogels and foams have shown the honeycomb-like morphology with cell dimension tens of micrometers and completed ultralow density as light as air^23^,^24^. Nevertheless, in these cell walls with several tens nanometer thickness, the extraordinary mechanical properties of individual carbon nanotube could not be harnessed effectively under compression. Once the inelastic collapse occurs, the weak interconnection between adjacent cell walls would cause poor mechanical stability and recovery performance under large-strain deformation^24^. In addition, the strength to density ratio is relative low in these 3D architectures due to their micrometer scale cell dimension. Thus, efficient assembly of CNT-based cellular solids with appropriate structure is the key to fully realize the potential of individual nanotubes in macroscopic architecture and achieve superb mechanical properties and stability. A hierarchical network like a 3D truss, proven to be highly beneficial to maximize bulk-specific elastic modulus and mechanical stability, has been widely utilized in engineering constructions and structural design of material. In our previous work, similar truss-like structure was achieved in macroscopic carbon nanotube monolithic sponges by chemical vapor deposition (CVD), in which individual nanotubes are randomly interconnected into 3D skeletons^25^,^26^,^27^,^28^,^29^,^30^,^31^. While earlier works have demonstrated the multifunctional properties of such CNT sponges, no comprehensive studies addressing their collective mechanical behavior have been reported yet. A thorough understanding of the mechanical response of this CNT-based structure to deformation will provide insight into their lifetime and sheds further light on structural design of nano-carbon material based 3D architectures.

In the present work, we performed a systematical structure-property study on macroscopic sponges of randomly interconnected carbon nanotubes grown via CVD. Transmission electron microscopy (TEM) characterization indicated the strong inter-tube bonding between neighboring nanotubes (junctions) in CNT sponges guarantees the reversible deformation without structural collapse under compression. Systematic mechanical tests indicated that the resulting CNT sponges could exhibit a combination of super-elasticity, fatigue resistance, thermo-mechanical stability and electro-mechanical stability, which cannot be observed in conventional polymer foam. Based on *in-situ* scanning electron microscopy (SEM) observation and nanotube deformation analysis, a theoretical mechanical model based on elastic theory was proposed to thoroughly describe the compressive behavior of spongy CNT, and was consistent well with experimental results. The detailed structure-mechanical analysis at microscopic level proposed in this work is helpful to not only clarify the origin of the mechanical deformation of 3D carbon materials, also develop a basis for structural design and optimization of nanostructured materials based 3D architectures.

## Results

CNT sponges, a sponge-like 3D solids synthesized by a chemical vapor deposition (CVD) method, have been reported in recent publications and have shown promise for environmental applications, smart materials and nanocomposites^25^,^26^,^27^,^28^,^29^,^30^,^31^. All samples were pretreated mechanically before further characterization in order to eliminate ‘preconditioning’ behavior^8^. The sample can be compressed dramatically without damage and will return to original position after released. Earlier works have indicated that bulk thickness, density and porosity of the macroscale CNT sponges with nanoscale pores could be directly controlled by growth time and source injection rate^25^. The bulk density of the samples in this work were measured to be ~15 mg/cm^3^ (compared to low density carbon aerogel of more than 4 mg/cm^3^)^23^.

**Figure 1:**
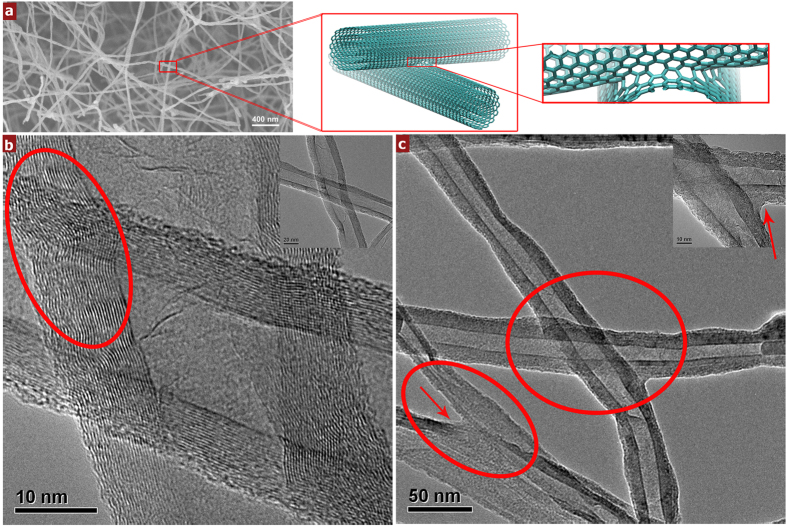
High-resolution SEM and TEM of the microstructure of carbon nanotube sponges. (**a**) SEM images of the 3D truss-like network. (**b**) High- and low (inset)-magnification TEM images of X-junction of CNTs with a representative schematic image in 1a. Red circle highlights the curved nanotube walls caused by chemically covalent interconnection. (**c**) High (inset)-and low-magnification hinge-like Y- and X-junction CNTs with a representative schematic image. Amorphous carbons around nanotubes are marked with red arrows.

Figure 1 shows the typical hierarchical truss-like microstructure of the synthesized 3D CNT sponge. Among them, the individual nanotubes with diameters ranged from 30–40 nm are randomly orientated. In general, creating junctions between neighboring CNTs is one of the most crucial steps needed to successfully synthesize CNT based 3D macroscopic architectures exhibiting superior material properties^12^,^32^,^33^,^34^. The connection stability between neighboring CNTs predominantly affects the compressive stability of their assembled macroscopic sponges. Recently, there have been a few efforts to build such covalently interconnected nanotube building blocks using the boron-doping^34^, graphene coating^9^, and chemical cross-linking^12^,^33^. In our case, the formation of a junction between neighboring two CNTs might be the result of continuous energy minimization during the growth process^35^,^36^,^37^. TEM is used to obtain more detailed morphological information of the CNT junctions in our materials. Obtained bright-field images further confirm the presence of the CNT junctions, shown in Fig. It is found that at the junctions, the nanotube walls become curved and wavy caused by chemically covalent interconnection (see Fig. 1a) or the amorphous carbon aggregation cause hinge-like junctions (see Fig. 1b) in our sponges. Their representative schematic figures are shown in Fig. 1a. CNTs with more complex junctions and the difference between connected and unconnected nanotubes in physical properties were characterized and shown in Supplementary Figure S1. Thus, in a sponge, strong interconnection held CNTs together and made them randomly overlap onto each other, leading to an isotropic network consisting of slender elastic tubes, forming a 3D truss. The strong binding force between neighboring nanotubes would guarantee the reversible deformation of elastic tubes such as bending and buckling without structural collapse under compressive loading.

**Figure 2:**
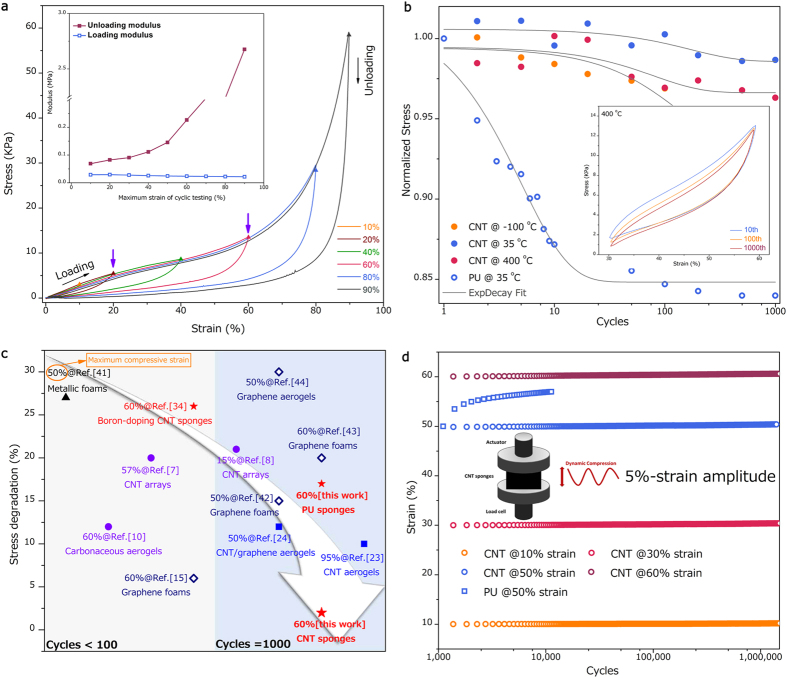
Compressive mechanical property and stability characterizations. (**a**) Loading and unloading compressive stress-strain curves of nanotube sponges at different set strains of 10, 20, 40, 60, 80 and 90%, respectively, showing that the area of the hysteresis loops increase at larger strain. Inset: measured loading (blue) and unloading (red) modulus with respect to maximum strain of the cycles. (**b**) Measured compressive stress response at the strain of 60% with respect to number of cycles. The colored filled and empty circles are the experimental data, a black line is the best fitting line for the data using the first order exponential decay function. Inset: Compressive cyclic testing of CNT sponges at 30%–60% strain, 0.016 Hz, 400 °C, for the 10^th^, 100^th^ and 1,000^th^ cycles. (**c**) Comparison of the relaxation properties of CNT sponges and other materials. Note that the stress degradation of other materials would be different with different densities or loading directions (anisotropy) and we chose the least value (best performance) as their relaxation value. (**d**) Fatigue strain-time for the CNT and PU sponges. Inset: schematic of compressive cyclic testing. Tests are conducted at room temperature, a strain amplitude of 5%, a test frequency of 50 Hz, for the CNT sponges at different set strains of 10, 30, 50 and 60% and PU of 50% strain only.

A thorough understanding of the mechanical response of CNT-based macroscopic sponges to deformation will provide insight into their structure-properties relationships. We focus mainly on their performance in mechanical stability such as fatigue resistance and long-term load bearing capacity. Here we first measured their compressive stress as a function of strain as shown in Fig. 2a. Curves obtained during the loading process show the three characteristic deformation regions typically observed in open-cell foams and bio-cellular materials^1^. A linear region for strain ≤ 20% with a Young’s modulus of ~ 0.025 MPa records the elastic bending of nanotubes, elastic buckling of nanotubes is recorded by a plateau region with gradually increasing slope after plateau strain (20%) and a densification region for strain > 60% with steeply rising stress^24^. While conventional open-cell foams displayed permanent deformation under moderate strains, CNT sponges exhibit intriguing structural stability, with nearly full recovery from large strains (90%) under uniaxial loading due to the elasticity of individual building blocks and strong inter-tube junctions. In Fig. 2a inset, loading-unloading cycles at various set strains of our samples show nearly similar loading linear regions, which indicate negligible degradation of the mechanical strength. In comparison, polyurethanes (PU) sponges were chosen as our benchmark in terms of mechanical stability because it is the commonest material with desirable physical properties used as commercial sponges, medical devices and biomaterials^38^.

As compressive mechanical stability performances of sponges or cellular solids at linear region and especially at plateau region are crucial for their engineering applications^1^, we further characterize their mechanical behavior before 60% strain (densification strain). Mechanical stability tests of the CNT sponges were conducted by cyclic compression at 0.016 Hz, 400 °C. Inset in Fig. 2b shows identical stress-strain behavior of CNT sponges after 1000 cycles at 60% strain with little stress degradation, indicating the excellent mechanical stability of the CNT sponges at high temperature. Identical stability was also observed at −100° and 35 °C, as evidenced by similar cyclic behavior (Supplementary Figure S2). This phenomenon displays excellent thermo-mechanical stability of our CNT sponges in wide temperature range: not only mechanical stability over many cycles but also the temperature-invariant mechanical performances. Comparatively, this superb stability cannot be observed in conventional PU sponges. The PU materials show urethane bond decomposition at about 260 °C and the degradation of polyol backbone at about 400 °C^39^. Moreover, once temperature decreased to −100°, the PU sponge became fragile and rigid, permanent deformation occurred during loading process. This is because polymer chains motion in polymeric materials is a thermally activated process, whereas the building block of our materials could hold its temperature-invariant mechanical properties^14^,^40^.

Different from elastic behavior of individual nanotube, the polymer chains motions, such as disentanglements, relocation and reorientation and so on, are viscoelastic and the viscoelastic behavior such as creep and stress relaxation would be detrimental to their service life and application ranges. Figure 2b shows the stress responses measured at the strain level of 60% as a function of the number of cycles for CNT and PU sponges (Supplementary Figure S2). Apparent stress relaxation behavior can be observed for PU sponges: the stress response gradually decreased and became stationary, reaching a constant. Generally, the relaxation behaviors would significantly influence the long-term durability of sponge materials. After 1000 compressive cycles, there is 17% stress degradation for PU sponges at 35 °C. In comparison, CNT sponges showed excellent elasticity and stress relaxation resistance as the degradation at −100, 35 and 400 °C is only 5%, 2% and 3%, respectively, highlighting their temperature-invariant mechanical stability performance.

Figure 2c shows a comparison of the relaxation properties of CNT sponges and other recently reported 3D sponge-like materials, including metallic foams^41^, CNT arrays^7^,^8^, carbonaceous aerogels^10^, CNT aerogels^34^, graphene foams and aerogels^15^,^42^,^43^,^44^, graphene and CNT/graphene hybrid aerogels^24^, under cyclic loading. In fact, the CNT sponges compare well with all other sponge-like solids in Fig. 2c^ ^7,8,10,15,24,34,41,42,43,44,45. It is worth to note that all CNT based cellular solids do not necessarily show super-elasticity though their elastic building blocks. Note that the word “super-elasticity” used in the field of mechanical characterization of nanomaterial-based assemblies, in general, literally refers to the strong capacity of recovery of materials, especially when undergoing large deformation. We attribute the outstanding load-bearing capacity to three significant advantages of our sponges: 1) Compared to other metals, polymer and even graphene building blocks, the carbon nanotubes build blocks in our materials are superelastic, allowing complete recovery after large deformation without plasticity, damage and fatigue^18^. 2) The nanoscale hierarchical microstructure of our materials leads to an isotropic 3D truss-like network consisting of individual elastic nanotubes, allowing independent deformation of each nanotube without entanglement which was typical observed in CNT arrays because of compressive mechanical instability of aligned forest structure^7^. Furthermore, each nanotube would deliver bending or buckling deformation and transfer the force under compression of the microstructure, and hence true mechanical elasticity and strength of individual carbon nanotube would be realized effectively in bulk sponges. For instance, at density level of ~5 mg/cm^3^, the Young’s modulus could be to as high as ~40 kPa^25^, much higher than that of PU sponges (3.7 kPa), graphene foams ( < 20 kPa)^42^, CNT aerogels (1.2–10 kPa, anisotropy)^23^. 3) Strong molecular level inter-tube connection includes chemically covalent junctions and the amorphous carbon aggregation caused hinge-like junctions, guaranteeing the large and reversible deformation of the nanotubes between connections under lager deformation compression without structural collapse. In addition, compressive cyclic testing with strain amplitude (5%) at 50 Hz were employed to assess the fatigue performance of CNT and PU sponges at various applied strain levels for at least 1.8 × 10^6^ cycles (Fig. 2d). In PU materials, about 7% fatigue strain (shrinkage from the original length) was observed after 10 thousand cycles at set strains of 50%, and there is no observable ceiling to this fatigue behavior, which could be considered as fatigue failure or degradation. Note, however, that the fatigue performance of CNT sponges is remarkably stable and only 0.35% fatigue shrinkage could be measured after enduring millions of cycles in Supplementary Figure S3, highlighting their structural robustness and fatigue resistance. Here the fatigue shrinkage of CNT sponges unlikes what is typically seen in conventional open-cell materials. The slight fatigue might be the result of negligible structural reorientation of the collective nanotube sponges system, rather than fracture or failure of individual nanotubes. By assuming 10% strain shrinkage to be a sign of fatigue failure, the life cycles of our CNT sponges could be estimated as more than 10^8^ at 60% strain, in stark contrast to that of PU samples (10^4^ at 50% strain), would be comparable to that of human skeletal muscle (10^9^), showing their potential use in synthetic biomaterial fields^8^,^46^.

**Figure 3:**
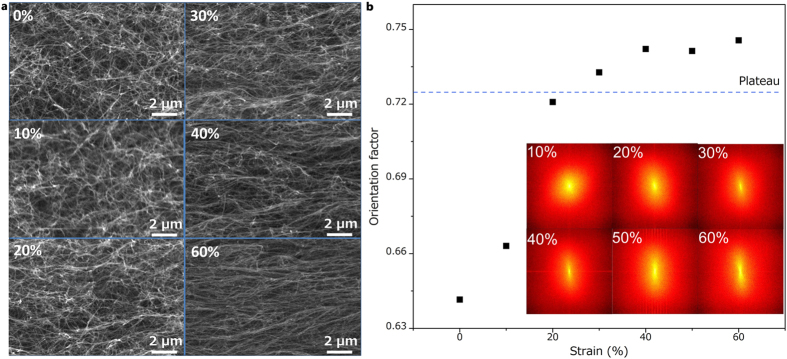
Microstructural evolution of carbon nanotubes sponges under compression. (**a**) SEM imaging normal to the compression direction, showing an isotropic orientation of microstructure within the sponges at 0% strain and an increasing alignment with increasing strain. (**b**) Orientation factor (*OF*) as a function of compressive strain. Insets: 2D FFT of the SEM images at various compressive strain.

In order to provide insight into the mechanism of the mechanical stability and further develop structure-property analysis to guide for design of nanocarbon-based cellular materials, we characterized the mechanical properties of the CNT sponges by determining the structural evolution of 3D truss-like network under compression strain via *in situ* SEM imaging normal to the load direction (Fig. 3a, more details in Supplementary Figure S4). Zooming in on a single unit cell of the truss reveals that the nanotubes with a small initial curvature constructed the cell structure. Under compression, the bending of horizontal-layout CNTs and buckling of vertical-layout CNTs in the cell are activated successively, causing structural evolution and partial orientation of CNTs normal to the compression direction. Beyond 20% compressive strain, buckling deformation gradually dominates the evolution of the CNT cell, horizontal-layout CNTs stop bending and a highly aligned structure is presented at 60% strain in Fig. 3a. This interesting structural evolution process is also demonstrated through calculating the orientation factor (*OF*) (Fig. 2d) from the fast Fourier transform (FFT) of SEM images in Fig. 2b insets. Note that *OF* = 0.5 means that the nanotubes are randomly oriented, and 1 is perfectly aligned. Up to 20% strain, the randomly aligned CNTs structurally deformed into aligned CNTs normal to the compression direction, and the *OF* steadily increased from 0.64 to 0.72. Beyond 20% strain, the *OF* plateaued, indicating little increase in alignment, which is caused by the freezing of the movement of horizontal-layout CNTs. Meanwhile the curvature of vertical-layout CNTs increases significantly compared to that of the initial morphology without any strain (more details in Supplementary Figure S4).

**Figure 4:**
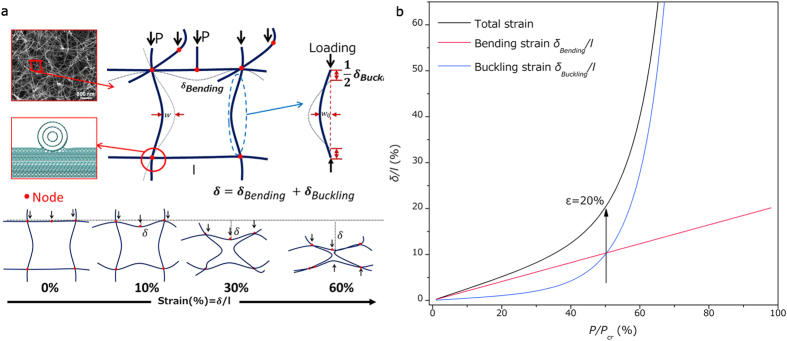
Microstructural evolution model. (**a**) Schematic description of the unit cell model of the CNT sponge and the evolution in cell structure with strain. Insets: SEM image of truss-like structure at 0% strain and schematic image of inter-tube junction (node). (**b**) Evolution of total strain, bending strain and buckling strain while the carbon nanotube cell is experiencing increasing compressive load. The total strain is 20% when the buckling strain began to exceed the bending strain.

On the basis of SEM observation of the structural evolution of CNT sponges, the unit cell model was taken out and shown in Fig. 4a, in which the cell consists of four slender nanotube beams. Within our model, *δ* is the compressive displacement and may be expressed as: *δ* = *δ*_*Bending*_ + *δ*_*Buckling*_ (Supplementary Figure S5). The first contribution, due to bending of horizontal-layout nanotubes, is computed from the linear-elastic deflection of a nanotube beam loaded at its midpoint by a load *P*. When a uniaxial stress is initially applied to the sponge so that each cell node (inter-tube junction) transfers the force, the nanotubes itself bends and shows elastically linear load-displacement relationship (Fig. 4b red line). Under increased strain, the second contribution, due to nonlinear but still elastic buckling of vertical-layout nanotubes, gradually increased its proportion in total strain (Fig. 4b blue line and Supplementary Figure S6), and eventually total stress-strain response entered the non-linear region^47^. Beyond a critical strain where bending and buckling strain contributed the total strain equally, a transient decrease in the load-strain slop (linear-plateau region transition in stress-strain curve) can be observed, indicating the elastic structural evolution of the unit cell structure (Fig. 2b black line). Meanwhile, at high strain level, the densification of the nanotube structure would result in increase of the modulus. The structural evolution model agrees with the compressive stress-strain curves of nanotube sponges in Fig. 2a and structural evolution process observed in SEM images in Fig. 2c. Thus, due to the elasticity and the strong molecular level junction between building blocks, the plateau strain for CNT sponges, unlike that for polymer sponges and conventional cellular solids, is a transformation from linear, elastic bending-dominated mode to nonlinear, still elastic buckling-dominated mode without any plastic deformation or structure collapse. Furthermore, different from that of aerogels and arrays, the carbon nanotubes of our sponges bend and buckle in 3D truss-like structure individually and are constrained by the strong junction connections in molecular level, allowing CNT sponges to be heavily compressed with outstanding structural and mechanical recovery.

We further could predict the experimental data such as plateau strain of our macroscopic CNT sponge through estimating the critical strain (bending-buckling-transition strain) of the microstructural evolution model. Based on the SEM and TEM results in Fig. 1, the outer diameter of CNT, the inner diameter, the length of the unit cell and initial curvature of nanotube *w*_*0*_*/l* was estimated as 33 nm, 16 nm, 500 nm and 0.1, respectively. After estimating the elastic modulus of CNT as 1 TPa, the calculated critical strain was 20.3% and agreed well with the experiment, further demonstrating that their excellent fatigue resistance and stress relaxation resistance stemmed from the structural features caused unique microstructural evolution behavior of CNT sponges. Furthermore, based on our model, the junctions distance, initial curvature and the mechanical properties of the junctions and nanotubes directly affect the microstructural evolution process, and hence future works could be guided for altering the CNT sponge’s microstructure, microstructural properties and, in turn, its bulk properties by density controlling^25^, chemical modification^34^, electron irradiation^48^ and graphene coating and so on^9^. Meanwhile, our thorough understanding of the mechanical response of the truss-like structures of our CNT sponges to deformation could also develop a basis for potential applications.

**Figure 5:**
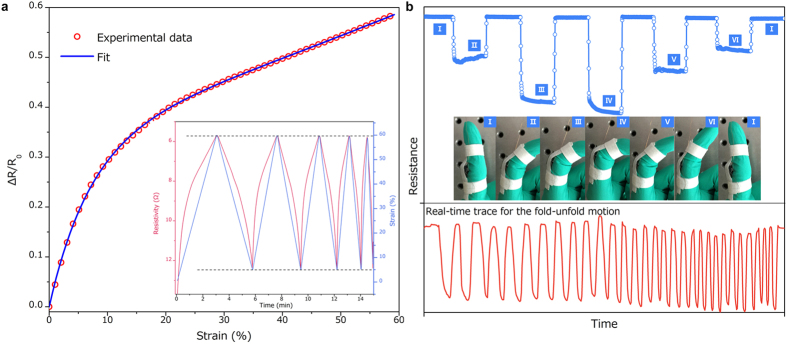
Electro-mechanical stability of CNT sponges. (**a**) Relative change in resistance versus strain. The resistivity measured on sponges was ~60% change at a strain amplitude of 60%, and resistivity-strain relationship became near linear and showed no observable ceiling to the changes in resistance. Inset: at the 5–60% strain range, the electrical response corresponding to cyclic loading with increasing frequency. (**b**) Demonstrations of using the sponges to detect human motion. Upper: corresponding resistance responses to finger motions in the insets (photograph of the finger during folded-unfold motion). Lower: real-time trace of the cyclic finger motion via CNT sponges.

The superelastic carbon nanotubes construct CNT sponges not only with excellent mechanical properties but also with high electrical conductivity, thus holding great potential for applications in flexible and compressible conductors and sensors^6^. Herein, in addition to monitoring the stability performance under cyclic compression, we also measured the strain dependence of electrical resistance (electro-mechanical stability) of the nanotube sponges to validate the feasibility and validity of them as smart sensors. As shown in Fig. 5a, the normalized electrical resistance (Δ*R/R*_*0*_) decreases dramatically with an increase in strain to ~20%, and then shows a near linear relationship. The gauge factor (ratio of normalized electrical resistance to its deformation) would be about 3 at low strain level, higher than that of graphene foam (~1.3) reported in our previous work^15^. Furthermore, due to the super-elasticity of spongy nanotubes, the cyclic loading with increasing frequency test (Fig. 5a inset) shows constant electro-mechanical properties (resistance at 60% and 5% strain). This indicates that nanotube architectures could survive the different loading frequency of electromechanical devious and survive without electrical signal distortion. Therefore, this combination of truss-like microstructure, excellent stability and electro-mechanical stability indicates that such engineered nanotube architectures could be used in intelligent structures without any damage. Herein, we present a straightforward application to demonstrate the potential of the CNT sponges for use in real-time human-motion detection. The CNT sponges sample was attached around a rubber glove to detect the bending movements of the fingers. When the demonstrator’s fingers were gradually folded, pressure would be exerted on the sponges and caused compressive deformation and hence a decrease in resistance of the sponges (Fig. 5b), allowing the detection of the folded amplitude of fingers. Due to their electro-mechanical performance as above-mentioned, the resistance of the sponges could return to the initial level once the fingers were completely unfolded. Notably, apart from tracing the amplitude of fingers’ motion, the frequency was easily detected simultaneously as shown in the real-time trace for the cyclic fold-unfold motions with increasing frequency in Fig. 5b. Hence, the use of such kind of spongy sensors might benefit both daily and engineering activities, such as health monitoring and remote controlling.

The complex structure of the macroscopic assemblies of carbon nanotubes leads to highly interesting piezoresistive performance of this new type of smart materials. Here, we further present an in-depth study of the piezoresistive effect in terms of structural deformation. Tunneling conduction theory was used to explain the resistance change in the nano-carbon based materials at low compressive strain levels^28^, while it cannot fit well with the nonlinear electromechanical coupling results at large strain region. Based on the microstructural evolution model as mentioned earlier, the theory was modified according to the following considerations and assumptions and fitting results accord quite well with the experimental results (Fig. 5, detail in Supplementary Figure S6). 1) Strain transfer factor: in nano-carbon based macroscopic architectures, the strain of contact distance might be different with macroscopic compressive strain and hence the strain transfer factor should be considered into the theory. 2) On the basis of microstructural evolution model: beyond plateau strain, the buckling deformation would raise curvature of vertical-layout CNTs and hence increase ‘new’ unconnected junctions and conducting path, causing additional resistance reduction under large-strain compression. Therefore, we suggest that the reduction of electrical resistance is caused by which the deformation of the microstructure decreases the contact distance between connected nanotubes and creates new unconnected junctions synergistically. Once the load is removed, microstructure of CNT sponges returns to their pre-compressed configurations, allowing them to spring back to their original shape and resistance.

## Discussion

The extraordinary flexibility and strength of individual carbon nanotubes were fully realized in the macroscopic hierarchical CNT sponges. Different from conventional cellular materials, the CNT sponges clearly exhibit super mechanical properties and stability: super-elasticity, high strength to weight ratio, thermo-mechanical stability in wide temperature range, negligible stress relaxation under high strain, excellent fatigue resistance after more than 3.5 × 10^6^ cycles and frequency-invariant electro-mechanical stability under mechanical compression. A thorough understanding of microstructural features (strong junctions between nanotubes) and evolution (completely elastic bending-buckling transition) of this CNT-based structure to deformation was proposed to clarify their mechanical properties and nonlinear electromechanical coupling behavior. Our work would guide for nanofiller-based cellular structure design and develop a basis for potential applications such as dampers, electrodes, electromechanical devices, synthetic biomaterials, nanocomposites.

## Methods

### Synthesis of CNT sponges

CNT sponges were synthesized by a CVD method reported by us previously^25^,^26^,^27^,^28^. Ferrocene powders (catalyst precursor) were dissolved in 1,2-dichlorobenzene to make a solution with a concentration of 60 mg/mL. In this case, dichlorobenzene was employed as a novel carbon source to disturb the aligned growth of the nanotubes, and thereby nanotubes were consecutively stacked in a random manner to form an interconnected 3D truss-like structure. The solution was pumped into the CVD furnace equipped with a quartz tube at a speed of 0.13 mL/min. A mixture gas flow of Ar (2000 mL/min) and H_2_ (300 mL/min) was used as the carrier gas. The growth temperature was fixed at 860 °C and the growth time was 4 h when the sponge reaches a thickness of about 8 mm. A piece of quartz glass placed in the middle of the quartz was used as the substrate where CNT sponges deposited.

### Structural and electrical characterization of sponges

The microstructure and morphology of the as-prepared sponges were characterized by SEM (HITACHI S3400). To give an insight of inter-tube structure, TEM (FEI Tecnai G2 F20 U-TWIN) observations were conducted directly on as-prepared samples. Thin CNT sheets were carefully separated from the CNT materials and directly deposited between two TEM grids to observe their initial inter-tube structure. For the electromechanical tests, the top and bottom surfaces of the CNT sponges were coated with a uniform layer of silver paste and connected by silver wires. During the compression process, the electrical resistance (Keithley 4200 SCS under a bias of 10 mA) was recorded simultaneously.

### Mechanical testing

A dynamic mechanical analyzer (TA, DMA Q800) was used to evaluate the mechanical performance of these sponges. The dimensions of the tested samples were about 0.6 cm × 0.6 cm × 0.4 cm for CNT sponges cubic and for 1.3 cm in diameter × 0.5 cm in height for PU spongy cylinder. All samples were pretreated by 100-time 30%–60% strain compression before all mechanical testing to eliminate the ‘preconditioning’ behavior observed in CNT-based assemblies^8^. All the samples were applied an initial load of around 0.05 N in order to provide uniform contact. Static compression tests in Fig. 2a were conducted in the strain ramp mode with a ramp rate of 10% min^−1^. Cyclic strain controlled loading was used to evaluate the fatigue behavior of the CNT and PU sample and the test frequency in Fig. 3a,c was 0.016 Hz and 50 Hz, respectively.

## Additional Information

**How to cite this article**: Dai, Z. *et al*. Three-dimensional Sponges with Super Mechanical Stability: Harnessing True Elasticity of Individual Carbon Nanotubes in Macroscopic Architectures. *Sci. Rep*. **6**, 18930; doi: 10.1038/srep18930 (2016).

## Supplementary Material

Supplementary Information
