# Supplementary material for: Three-dimensional Sponges with Super Mechanical Stability: Harnessing True Elasticity of Individual Carbon Nanotubes in Macroscopic Architectures
Source: Sci Rep. 2016 Jan 6;6:18930. doi: 10.1038/srep18930 (PMC4702113; doi:10.1038/srep18930)
Supplement: Supplementary Information [file srep18930-s1.pdf]

SUPPLEMENTARY INFORMATION

Three-dimensional Sponges with Super Mechanical Stability: Harnessing True Elasticity of Individual Carbon Nanotubes in Macroscopic Architectures

Zhaohe Dai[1,3], Luqi Liu*[1], Xiaoying Qi[1], Jun Kuang[1],Yueguang Wei[3], Hongwei Zhu*[2], Zhong Zhang*[1,2]

[1]CAS Key Laboratory of Nanosystem and Hierarchical Fabrication, National Center for Nanoscience and Technology, Beijing, 100190, P.R. China

[2]Center for Nano and Micro Mechanics (CNMM), Tsinghua University, Beijing 100084, P.R. China

[3]State Key Laboratory of Nonlinear Mechanics, Institute of Mechanics, Chinese Academy of Sciences, Beijing, 100190, P.R. China

S1 Microstructure of connected and unconnected nanotubes.

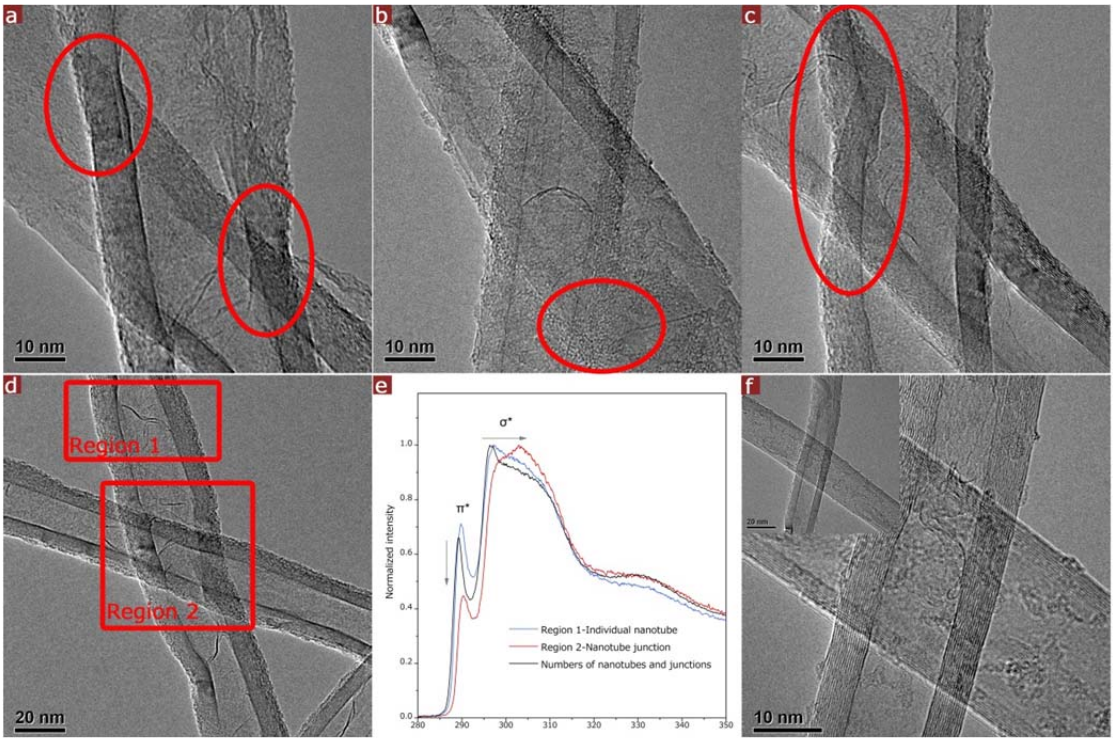

Figure S1. (a-d) High-resolution transmission electron microscopy of the inter-tube structures. connected CNTs with complex junctions as highlighted by the red circles. (e)Electron energy loss spectroscopy (EELS) spectra, confirming difference between the connected junctions and individual tubes in sp^2 bonds. The black curve exhibits the overall state of the whole materials. (f) The inserted image shows the two unconnected nanotubes, in which the walls at the 'junction' are as straight as highways, quite different with that at connected junction in Figure 1c. In fact, the scenario of unconnected nanotubes is really rare that this figure was found after searching of tens of connection (a quantitative possibility is about 5%), and we hence suggest that near all individual CNTs are connected each other in our CNT sponge system.

S2 Mechanical properties of the CNT materials across -100 °C to 400 °C

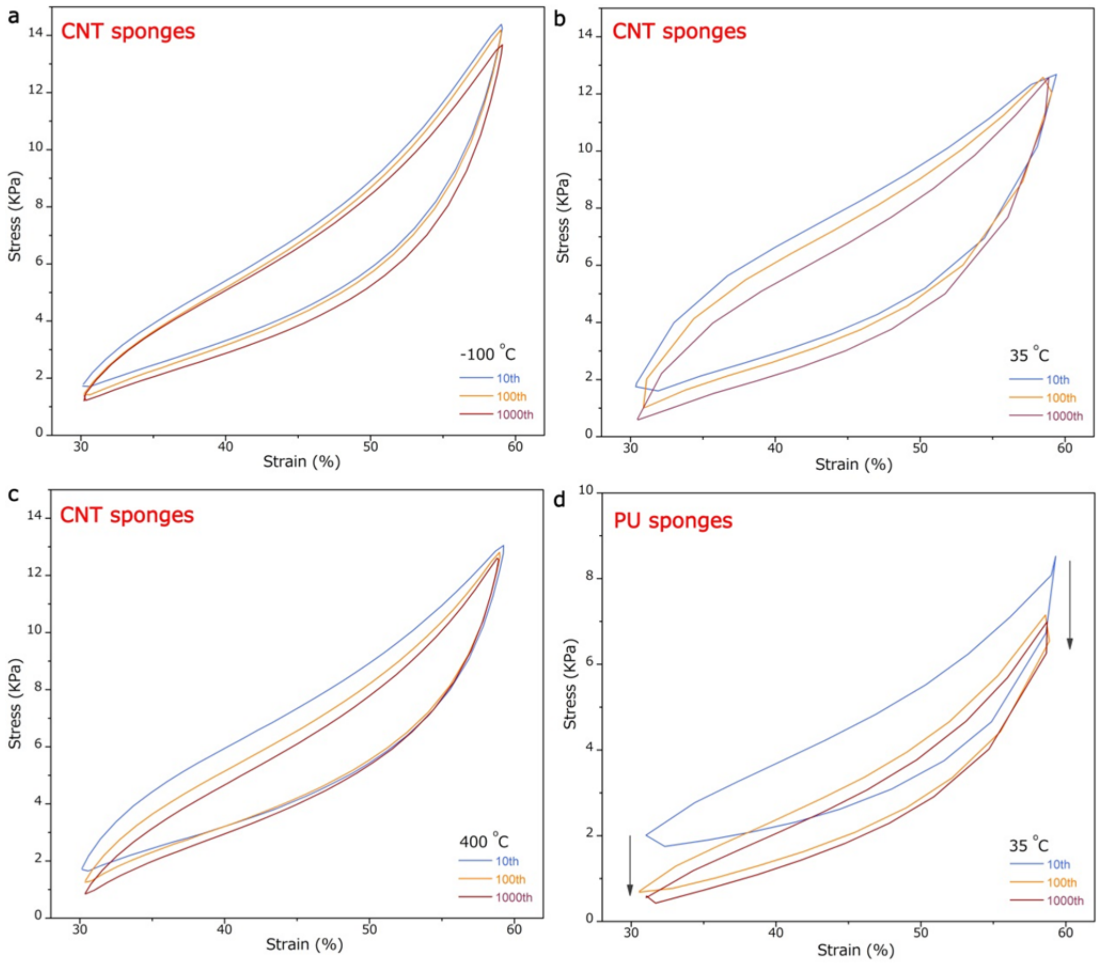

Figure S2. Mechanical properties of the CNT and PU materials. Cyclic test (30%-60% strain, 0.016 Hz, 1000 cycles) at (a) -100 °C, (b) 35 °C , (c) 400 °C for CNT sponges and (d) 35 °C for PU sponges.

S3 Strain-fatigue relation in cyclic strain controlled testing.

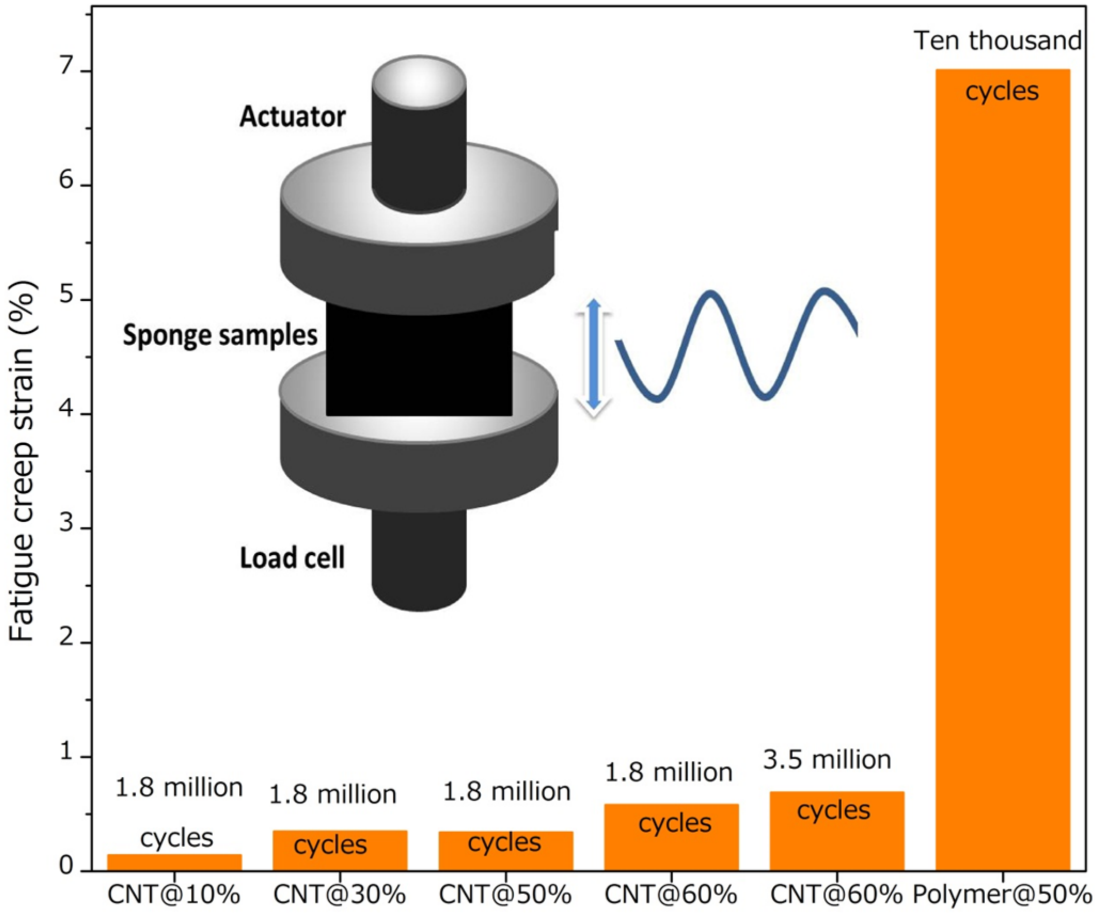

Figure S3. Fatigue strain-cycles for the CNT and PU sponges. Insert: schematic of compressive cyclic testing. Tests are conducted at room temperature, a strain amplitude of 5%, a test frequency of 50 Hz, for the CNT sponges at different set strains of 10, 30, 50 and 60% and PU of 50% strain only. In PU materials about 7% fatigue strain was observed after 10 thousand cycles at set strains of 50%. The fatigue strain in CNT sponges after 1.8 million cycles at various strains of 10, 30, 50 and 60% is 0.14, 0.35, 0.34 and 0.58%, respectively. Note that even after 3.5 million cycles, the CNT sponges showed only 0.7% fatigue shrinkage.

S4 SEM images at varying compressive strain and orientation factor calculation.

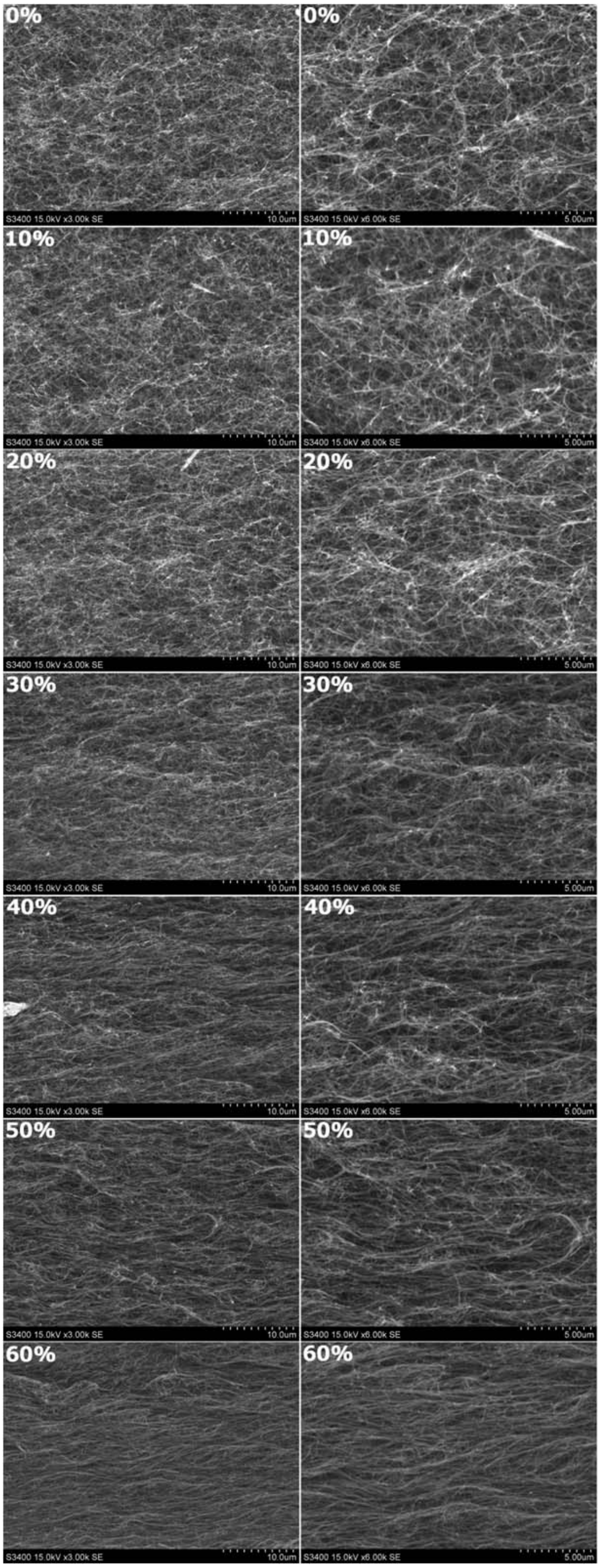

Figure S4. SEM images at varying compressive strain. FFT is performed on each SEM image, where the special image is converted to a frequency domain. As an inherent property of the FFT frequency domain images, the intensity of each pixel presents an angular dependence on patterns of spatial alignment where pixels displaying high intensity values are clustered along the orientation of the highest degree of directional anisotropy. Orientation Factor was calculated from the standard formulation as defined by $F = <cos^2\theta> = \frac{\int_0^\pi I_\theta cos^2\theta \, d\theta}{\int_0^\pi I_\theta \, d\theta}$, Where I is the intensity profile as a function of θ, and θ is the angle between the structural unit vector and the reference direction. In this case, the intensity profile was derived from FFT of side-view SEM images.

S5 Microstructural evolution model

Linear elasticity holds for small strains (<20%) and is controlled by two different strains: bending of horizontal-layout nanotubes and buckling of vertical-layout nanotubes. δ is the compressive displacement and may be expressed as: $\delta = \delta_{Bending} + \delta_{Buckling}$. Figure 3a shows the schematic diagram of the cell model of the CNT sponge. The first contribution, due to bending of horizontal-layout nanotubes, is computed from the linear-elastic deflection of a beam of unitary length loaded at its midpoint by a load *P*. When a uni-axial stress is applied to the foam so that each cell node transfers the force *P*, the nanotubes itself bends and the linear-elastic deflection: $\delta_{Bending} = \frac{Pl^3}{48EI}$. [Timoshenko, S. P.; Gere, J. M., *Theory of elastic stability*. Courier Dover Publications: 2009]

When a nanotube is submitted to the action of lateral load only, a small initial curvature of the tube has no effect on the bending and the final deflection curve is obtained by superposing the ordinates due to initial curvature on the deflections calculated as for a straight bar. However, if there is an axial force action on the bar, the deflections produced by this force will be substantially influenced by the initial curvature.

The initial shape of the axis of tube is given by the equation:

$$w_0(x) = w_0 \sin\frac{\pi x}{l}$$

Thus the axis of the tube has initially the form of a sine curve with a maximum ordinate at the middle equal to w_0. If this tube is submitted to the action of a longitudinal compressive force *P*, additional deflections w_1 will be produced so that the final ordinates of the deflection curves are $w=w_0+w_1$. Then the deflections w1 due to deformation are determined in the usual way from the differential equation:

$$EIw_1'' + P\left(w_1(x) + w_0(x)\right) = 0$$

Or by substituting initial shape equation for w_0 and using the notation $k^2=P/EI$, where *EI* is the bending stiffness of the nanotube beam, we obtain:

$$w_1'' + k^2 w_1 = -k^2 w_0 \sin\frac{\pi x}{l}$$

The general solution of this equation is:

$$w_1 = A\sin kx + B\cos kx + \frac{w_0}{\pi^2 / k^2 l^2 - 1}\sin\frac{\pi x}{l}$$

To satisfy the end conditions(w_1= 0, for x= 0 and x= l) for any value of k, we must put A= B= 0. Then, by using the notation α and critical load P_{cr}:

$$\alpha = \frac{P}{P_{cr} - P} = \frac{P}{\frac{\pi^2 EI}{l^2} - P} = \frac{1}{\frac{\pi^2}{k^2 l^2} - 1} \quad \text{and} \quad P_{cr} = \frac{\pi^2 EI}{l^2}$$

we obtain: $w_1 = \alpha w_0 \sin\frac{\pi x}{l}$. The final ordinates of the deflection curve are:

$$w = w_0(x) + w_1(x) = w_0 \sin\frac{\pi x}{l} + \alpha w_0 \sin\frac{\pi x}{l} = (1+\alpha) w_0 \sin\frac{\pi x}{l}$$

Then the elastic strain energy of the element could be calculated from the standard formula as defined by: $V = \int \frac{M(x)^2}{2EI} ds + \int \frac{P(x)^2}{2EA} ds \qquad ds = \sqrt{1 + w'^2}\,dx$, where EA is the compressive stiffness of the nanotube beam. According to the energy method which is expressed as $\delta_{Buckling} = \frac{dV}{dP} = \frac{\partial V}{\partial P} + \frac{\partial V}{\partial \alpha}\frac{\partial \alpha}{\partial P}$, the buckling displacement $\delta_{Bending}$, the total compressive displacement δ and the total compressive strain ε could be estimated as:

$$\delta_{Buckling} = \left[\frac{\alpha^2 w_0^2}{2EI}\left(1 + \frac{\pi^2 \alpha^2 w_0^2}{8l^2}\right) + \frac{1}{EA}\left(1 + \frac{\pi^2 \alpha^2 w_0^2}{4l^2}\right)\right] Pl + \left(\frac{w_0^2}{2EI} + \frac{\pi^2 \alpha^2 w_0^4}{8EIl^2} + \frac{\pi^2 w_0^2}{4EAl^2}\right)\alpha^3 P_{cr} l$$

$$\delta = \left[\frac{\alpha^2 w_0^2}{2EI}\left(1 + \frac{\pi^2 \alpha^2 w_0^2}{8l^2}\right) + \frac{1}{EA}\left(1 + \frac{\pi^2 \alpha^2 w_0^2}{4l^2}\right)\right] Pl + \left(\frac{w_0^2}{2EI} + \frac{\pi^2 \alpha^2 w_0^4}{8EIl^2} + \frac{\pi^2 w_0^2}{4EAl^2}\right)\alpha^3 P_{cr} l + \frac{Pl^3}{48EI}$$

$$\varepsilon = \frac{\delta}{l} = \left[\frac{\alpha^2 w_0^2}{2EI}\left(1 + \frac{\pi^2 \alpha^2 w_0^2}{8l^2}\right) + \frac{1}{EA}\left(1 + \frac{\pi^2 \alpha^2 w_0^2}{4l^2}\right)\right] P + \left(\frac{w_0^2}{2EI} + \frac{\pi^2 \alpha^2 w_0^4}{8EIl^2} + \frac{\pi^2 w_0^2}{4EAl^2}\right)\alpha^3 P_{cr} + \frac{Pl^2}{48EI}$$

In this eqution, we found that the strain-load relation is linear when the load is small (α<<1). The the nodes distance l, initial curvature w_0/l and the mechanical properties of nanotubes (EI and EA) directly affect this strain-load curves. Based on our model, the junctions distance, initial curvature and the mechanical properties of the junctions and nanotubes would affect the microstructural evolution process. For example, in Figure S5, with the increased initial curvature of the nanotube in the sponges, the critical strain

showed decreased tendency, indication that the stress-strain curve of the bulk sponges would enter the second regime more easily.

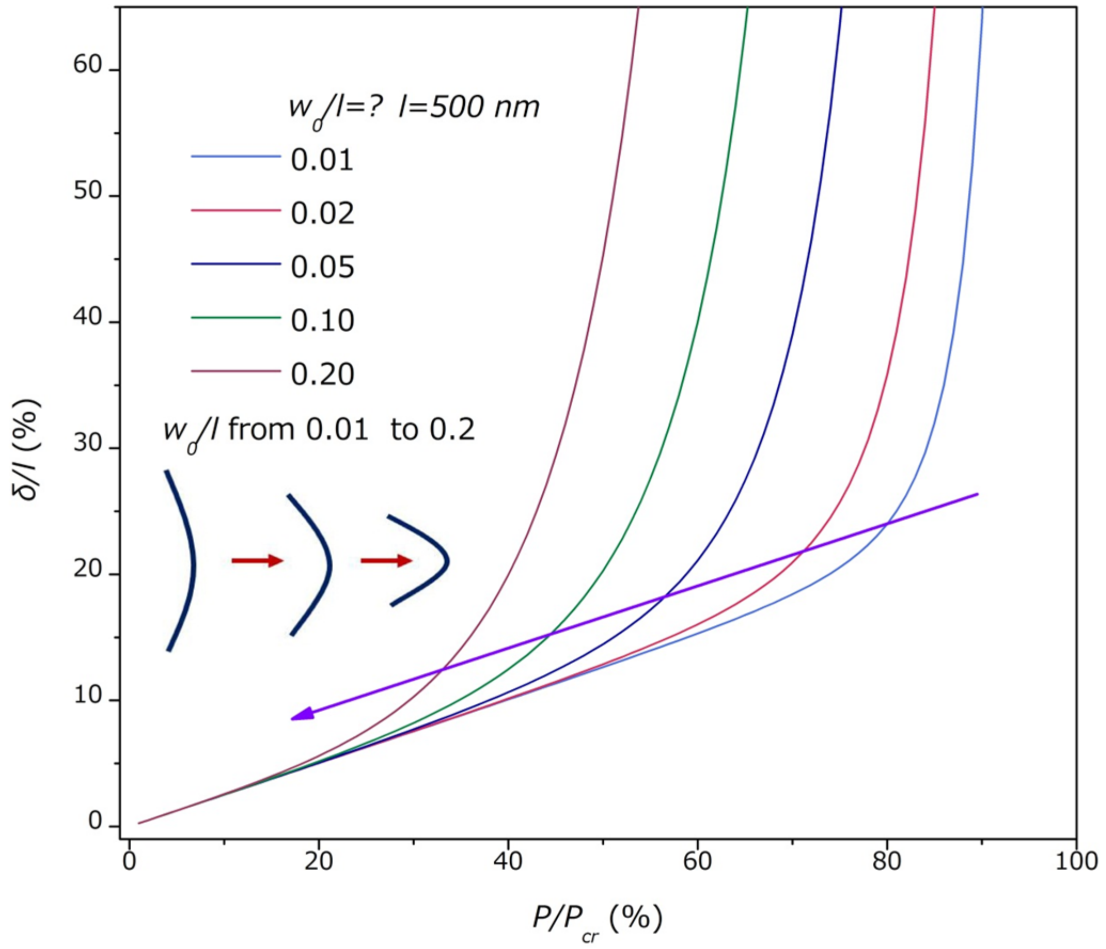

Figure S5. The evolution of the strain-load curves while the carbon nanotube network is experiencing compressive loads at different initial curvature values as annotated.

S6 Tunneling conduction theory

The electrical resistance of nano-carbon materials (NM) based macro-scale architectures mainly comes from two portions: NM itself and NM interconnections; and the latter contribute dominantly for the overall resistance. Hence, it has been proposed that the piezoresistive effect in nano-carbon materials is related to the contact resistance (*R*). *R* can be described based on a tunneling model. According to this model, the relationship between R and tunneling distance *d* can be derived as following:[Gui, X. et.al. *ACS nano* 2011, 5, 4276-4283.]

$$R = \frac{8\pi hL}{3a^2XdN}e^{Xl} \tag{1}$$

$$X = \frac{4\pi\sqrt{2m\varphi}}{h} \tag{2}$$

where h is Plank's constant; L is the number of particles forming a single conducting path; N is the number of conducting paths; *d* is the distance between conductive particles; a^2 is the effective cross-section area producing the tunneling effect; *e* and m are the electron charge and mass, respectively; and φ is the height of potential barrier between adjacent particles.

Under uniaxial compression, NM islands may move apart or closer to each other depending on specific configurations. Only NM islands with reduced distance could form new tunneling points and contribute to the conductivity enhancement. The distance between islands being compressed closer can be related to the compressive strain with a strain transfer factor α as:

$$d = d_0(1 - \alpha\varepsilon) \tag{3}$$

When the distance decreases from initially d_0 to an intermediate level of d during loading, correspondingly, the value of conduction resistance changes from initially R_0 to *R*. According to Eqs. (1) and (3), the relative resistance reduction due to tunneling conduction can be figured out by:

$$\frac{\Delta R}{R_0} = \frac{R_0 - R}{R_0} = 1 - (1 - \alpha\varepsilon)e^{-Xd_0\alpha\varepsilon} \tag{4}$$

In fact, due to the initial resistance (IR) of the NM itself and related equipment, the relative resistance reduction could not be 100%. After measuring the initial resistance, the theory-IR could be: $\frac{\Delta R}{R_0} = \frac{1-(1-\alpha\varepsilon)e^{-Xd_0\alpha\varepsilon}}{1+k}$ where *k* (0.5 in our system) is the ratio of the IR to the total resistance at 0% strain. In this work, we assume that the bending strain would results in tunneling conduction effect and the influence of buckling strain on resistance reduction is originated from the increased conducting path caused by the

raising curvature of vertical-layout CNTs. After assuming linear relationship between the buckling strain and resistance reduction to be *a*, the relative resistance reduction as a function of total strain can be calculated as:

$$\frac{\Delta R}{R_0} = \frac{1-(1-\alpha g(\varepsilon)\varepsilon)e^{-Xd_0\alpha\varepsilon}+(1-g(\varepsilon))\alpha\varepsilon}{1+k} \tag{5}$$

Where *g(ε)* is the function of the ratio of the bending strain to total strain with increasing total strain in Figure S6. The fitting results in our sponges show that the linear relationship between the buckling strain and resistance reduction is 1.14 ± 0.01 and changes at the distance between tubes can be 4.3 ± 0.6 times larger than macroscopic compressive strain.

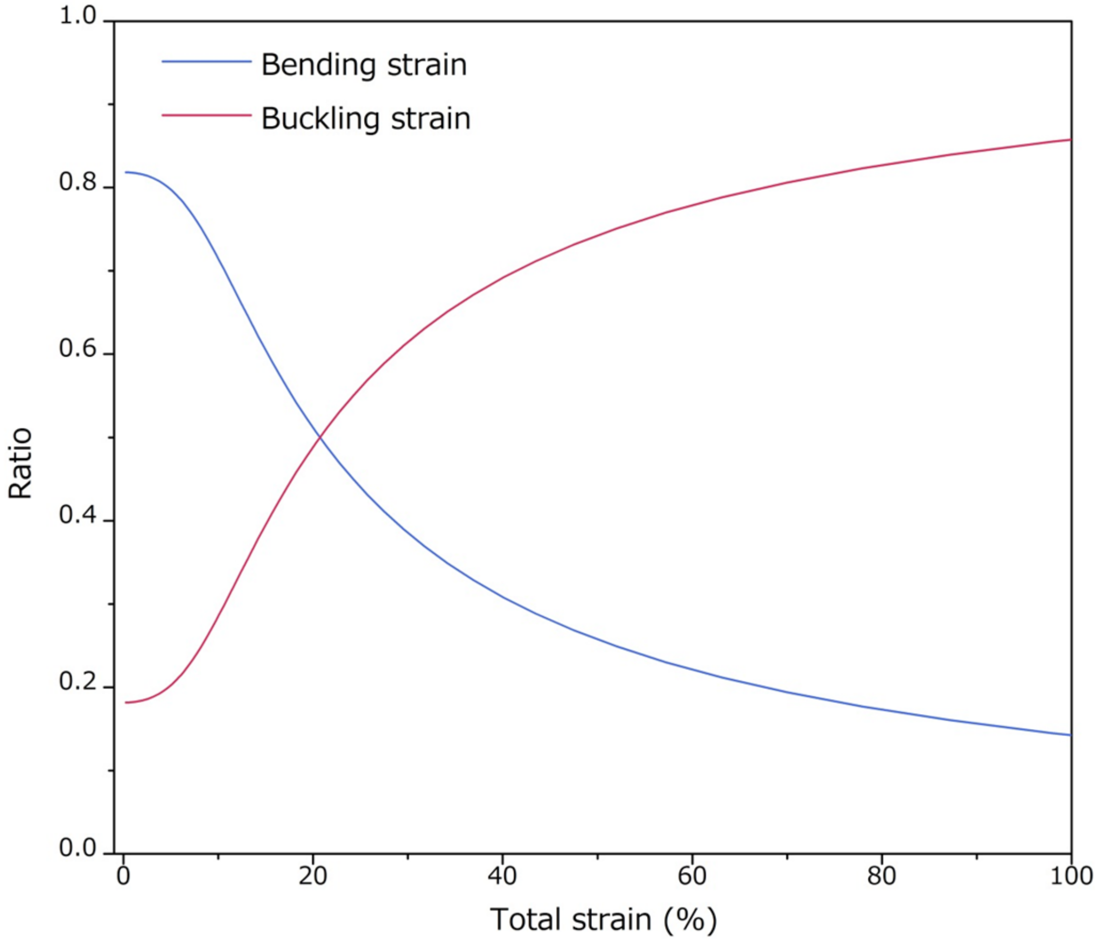

Figure S6 On the basis of the above-mentioned microstructural evolution model, the ratio of the bending strain (blue) and buckling strain (red) to total strain with increasing total strain from 0% to 60% could be calculated.
